# Maturation Process, Nutritional Profile, Bioactivities and Utilisation in Food Products of Red Pitaya Fruits: A Review

**DOI:** 10.3390/foods10112862

**Published:** 2021-11-18

**Authors:** Yanyi Huang, Margaret Anne Brennan, Stefan Kasapis, Samantha J. Richardson, Charles Stephen Brennan

**Affiliations:** 1 School of Science, Bundoora West Campus, RMIT University, Melbourne, VIC 3083, Australia; s3907309@student.rmit.edu.au (Y.H.); Stefan.kasapis@rmit.edu.au (S.K.); Samantha.richardson@rmit.edu.au (S.J.R.); 2 Department of Wine, Food and Molecular Biosciences, Lincoln University, Christchurch 7647, New Zealand; Margaret.Brennan@lincoln.ac.nz

**Keywords:** red pitaya fruits, polyphenols, betanin, polysaccharides, pectin

## Abstract

Red pitaya (*Hylocereus polyrhizus*, red pulp with pink peel), also known as dragon fruit, is a well-known species of pitaya fruit. Pitaya seeds and peels have been reported to exhibit higher concentrations of total polyphenols, beta-cyanins and amino acid than pulp, while anthocyanins (i.e., cyanidin 3-glucoside, delphinidin 3-glucoside and pelargonidin 3-glucoside) were only detected in the pulp extracts. Beta-cyanins, phenolics and flavonoids were found to increase gradually during fruit maturation and pigmentation appeared earlier in the pulp than peel. The phytochemicals were extracted and purified by various techniques and broadly used as natural, low-cost, and beneficial healthy compounds in foods, including bakery, wine, dairy, meat and confectionery products. These bioactive components also exhibit regulative influences on the human gut microbiota, glycaemic response, lipid accumulation, inflammation, growth of microbials and mutagenicity, but the mechanisms are yet to be understood. The objective of this study was to systematically summarise the effect of red pitaya’s maturation process on the nutritional profile and techno-functionality in a variety of food products. The findings of this review provide valuable suggestions for the red pitaya fruit processing industry, leading to novel formulations supported by molecular research.

## 1. Introduction

Pitahaya, or the pitaya fruit, is a well-known member of the Cactaceae family and is widely cultivated in tropical and subtropical areas [1]. Pitaya fruit is classified based on the colour of pulp and peel, namely white-pulp with pink peel pitaya (*Hylocereus undatus*), red-pulp with pink peel pitaya (*Hylocereus polyrhizus*) and white-pulp with yellow skin (*Hylocereus megulanthus*) [2]. Hua, et al. [3] reported that pitaya fruit is a good source of vitamins, dietary fibre, betacyanin, organic acids, amino acids and sugars. In the past decade, the utilisation of betacyanin (natural colourants) and pectin from pitaya fruit in food products has been studied. Evidence has reported that beta-cyanins (a type of betalains) derived from red pitaya fruit exhibit antidiabetic effects and modulate glycaemic response [4,5,6].

The high number of nutritional compounds in pitaya fruit has created interest in the food industry which regards to their use as economic, environmentally friendly, plant-based and clean-label ingredients [7]. Glucose, fructose and oligosaccharides are dominant carbohydrates present in red pitaya [8]. Additionally, polysaccharides and polyphenols are well-known antioxidants and can be used for prebiotic enrichment as well as natural colourants (betanin) in food products [9,10]. Essential fatty acids, especially linoleic acid, extracted from pitaya seeds have been reported to have laxative activity on gastroenteritis [11].

In this literature review, the maturation process of red pitaya and its functional nutrition that encompasses phenolic acids, flavonoids, betalains, polysaccharides, pectins and triterpenoids are discussed in relation to glycaemic modulation and usage in food processing.

## 2. Pitaya Species and Maturation

**Figure 1:**
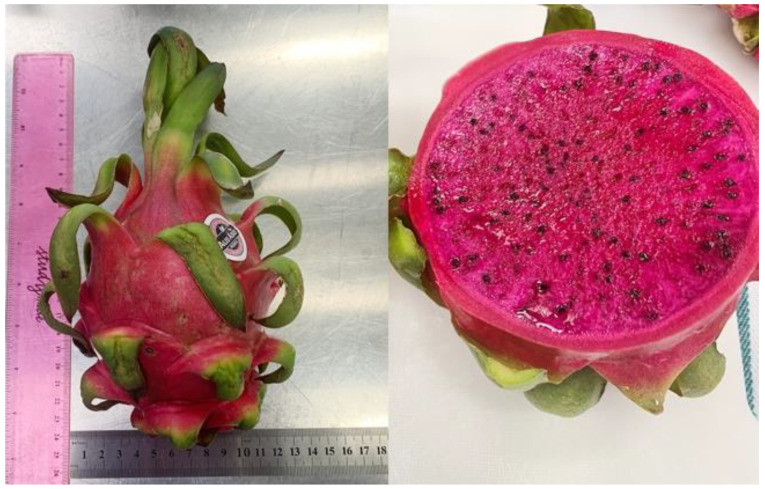
Red pitaya fruit.

Pitaya fruit (*Hylocereus*) or dragon fruit is indigenous in Mexico and Central America [12]. Its attractive colour appearance, low-calorie values and sweet flavour have been very attractive to the consumer. *Hylocereus* comprises 14 species, and they have been mainly classified by their skin and flesh colour. *Hylocereus undatus* (white flesh and pink skin), *Hylocereus polyrhizus* (red flesh and red skin, shown in Figure 1), and *Hylocereus megulanthus* (white flesh and yellow skin) have been the three most commonly consumed and studied pitaya species in recent years [13]. Pitaya fruit is highly perishable throughout postharvest storage due to the high moisture content present in the pulp. A study indicated that melatonin treatment effectively delayed fruit deterioration by reducing respiration intensity and oxygen radical production [14]. However, little information is known regarding the physicochemical changes that occur during fruit growth and maturation

### 2.1. Cultivars

Red pitaya is egg-shaped, weighs between 300–600 g and is between 13–15 cm in length [15]. White pitaya fruit has a spherical shape and tends to be larger in size than the red pitaya, reaching 300–800 g in weight and 15–22 cm in length [16]. Yellow pitaya (a medium-sized fruit weighing 180–250 g) has been reported to be a potential source of betaxanthins, beta-carotene, vitamin E and lycopene, which have been widely used as a yellow colourant in gummy confection, beverages and yoghurts [17]. More recently, a comparative study was conducted to analyse the bioactivity and cytotoxicity of white, red and yellow pitaya fruits [18]. Three phenolics, namely caffeic, gallic and protocatechuic acids, along with three flavonoids (myricetin, rutoside and quercetin), were identified and quantified in the study. Yellow pitaya had the highest levels of gallic acid and quercetin, while having the lowest amounts of rutoside, beta-cyanins and total polyphenols. Caffeic acid was the primary phenolic acid detected in white pitaya. Comparatively, red pitaya fruits exhibited the highest concentration of protocatechuic acid, total polyphenols, antioxidant activity and cytotoxic properties compared to that in both white and yellow pitaya fruits.

### 2.2. Fruit Growth and Maturation

Two red pitaya cultivars, namely ‘Guanhuahong’ (red pulp with red peel) and ‘Guanhuahongfen’ (pink pulp with red peel) and a white pitaya ‘Guanhuabai’ (white pulp with red peel) were studied by Hua, Chen, Zur, Wang, Wu, Chen, Zhang, Zhao, Hu and Qin [3]. It was reported that the chlorophyll contents decreased in the peel of both ‘Guanhuahong’ and ‘Guanhuahongfen’ cultivars during maturation, while the levels of betacyanin, betaxanthin, total phenols, flavonoids and antioxidant capacity dramatically increased in both pulp and peel when red pitaya fruits matured. Starch (376.7 g/fruit), sugars, including glucose (39.7%), fructose (29.3%), sorbitol, inositol, and sucrose (1.9%), and organic acids (malic, citric, citramalic and oxalic acids) gradually accumulated in the red pitaya pulp during maturation. Whereas the betacyanin content in white pitaya pulp remained stable, a significant decrease of betaxanthin and total flavonoid levels in the pulp were observed during fruit maturation.

In another study, the effect of maturation at nine different stages on beta-cyanins in red pitaya (‘Zihonglong’ cultivar) was investigated [19]. The study found that pigmentation appeared earlier in the pulp than peel, and the pulp colour turned into red within a day (on the 29th day) due to the sharp increase of betalain and the fading of green colour in the peel. Similarly, betacyanin and betaxanthin contents in both peel and pulp gradually, with some fluctuations, increased to 32.54 and 12.19 mg/100 g dry weight, respectively. Additionally, the study identified 65 metabolites and 51 were quantified in the fruits using chromatography, in which 15 amino acids (primarily amines, alanine, methionine, and phenylalanine) were identified in the red pitaya fruits. Results revealed that the red pitaya pulp exhibited higher amino acids, soluble sugars, and organic acids than the peel. Researchers also studied the correlation between metabolites and betalain formation using partial least-squares discriminant analysis and reported that citramalic acid had a strong correlation with the formation of betalain pigments. However, the mechanism of citramalic acid affecting betalain synthesis was not elucidated.

## 3. Nutritional Profiles and Health Benefits

### 3.1. Polyphenols

#### 3.1.1. Polyphenol Quantification

Polyphenols are a group of antioxidant compounds derived from plants, including phenolic acids, flavonoids, stilbenes and lignin, with high radical scavenging ability that reduces the risk of chronic diseases (cancers and cardiovascular problems) [20]. Polyphenols are reported to actively stimulate the growth of health-boosting microbes, including *Lactobacillus*, *Barnesville* and *Bifidobacterium*, in the human gut while impeding the growth of unhealthy microbes, e.g., *Escherichia coli* [5]. Polyphenols were found to inhibit α-amylase and α-glucosidase activity together with the modulation of postprandial hyper-glycaemia due to the interaction between starch and polyphenols [21]. Red violet beta-cyanins and yellowish betaxanthins are two common types of betalains that are rich in pitaya. These pigments have been reported to have high bioactive activity, and anti-inflammatory and anti-tumour cell line health benefits [22]. However, different extraction and quantification methods yield distinct polyphenol concentrations and profiles.

The use of different extraction methods, solvents and extracted conditions greatly impact on the concentration of phenolics, flavonoids and beta-cyanins. Table 1 summarises the different extraction and quantification methods used in recent research and the content of total phenolic acids, flavonoids and beta-cyanins in pitaya fruit. Ethanol (80%) and ultrasound-assisted extraction were employed by Arivalagan, et al. [23] to extract polyphenols in red pitaya. The content of total phenolic acids, flavonoids and beta-cyanins present in red pitaya pulp were about 48.30 mg Gallic acid Equivalent/100 g, 31.2 mg Citric acid Equivalent/100 g and 20.40 mg Betacyanin Equivalent/100 g, respectively. Higher results (2.39–10.14, 3.22–5.04 and 0.43–1.66 mg/g, respectively) were obtained in another study, in which optimal microwave-assisted extraction conditions of bioactive compounds in pitaya fruit pulp were investigated with the HPLC-DAD-ESI/MS and HPLC-DAD [24].

Pitaya seeds and peels possessed higher total polyphenols and beta-cyanins compared to pulps. Analysis of scientists analysed the optimized ultrasonic temperatures (30–70 °C), solvent to solid ratios (10:1–30:1), solvent concentrations (30–60% ethanol) and ultrasonic treatment times (5–25 min) and showed that the most efficient extraction yields of phenolics and betacyanin were obtained when the ratios of solid and solvent, temperatures and extraction times reached 25:1 g/mL, 60 °C and 20 min. Extract concentrations ranged from 4.51–8.54 mg GAE/g dry weight to 1.02–1.60 mg/g dry weight, respectively [2]. Red pitaya peel, pulp and seed were freeze-fired and phytochemicals were extracted in a hydroalcoholic solution at pH 2 followed by filtration and vacuum-drying [25]. Red pitaya seeds exhibited the highest amount of total phenolics and flavonoids, with 375.1 mg GAE/g and 264.4 mg Quercetin Equivalent/g, followed by peels, 294.8 mg GAE/g and 193.8 mg QE/g, whereas red pitaya peel contained less anthocyanin levels (135.4 mg Cyanidin chloride Equivalent/g) than that in the pulp (159.7 mg CCE/g).

#### 3.1.2. Chromatographic Identification

With the advancement of liquid chromatography coupled with mass spectrometry techniques, polyphenol profiles in red pitaya have been efficiently and accurately identified. A group of scientists analysed the composition of phenolic acids in red pitaya and reported that pitaya was composed of 11 types of phenolic acid: caffeic acid, ferulic acid, protocatechuic acid, p-coumaric acid, sinapic acid, vanillic acid, gallic acid, o-coumaric acid, t-cinnamic acid, salicylic acid and syringic acid; nine flavonoids: catechin, quercetin, epicatechin, epigallocatechin gallate, procyanidin B1, procyanidin B2, kaempferol 3-glucoside, quercetin 3-glucoside and rutin; and three anthocyanins: cyanidin 3-glucoside, delphinidin 3-glucoside and pelargonidin 3-glucoside [23,25,31]. These data are summarised in Table 2. The UPLC-MS/MS method was applied to identify the dominant phenolic acids, caffeic acid (20.1 mg/100 g) and ferulic acid (14.37 mg/100 g), present in red pitaya (*Hiriyur* species) [23]. Outcomes were consistent with the findings of García-Cruz, Dueñas, Santos-Buelgas, Valle-Guadarrama and Salinas-Moreno [16], who reported the highest concentrations for caffeoyl hexoside I and II (loss of a hexosyl moiety in caffeic acid).

Extracts of red pitaya pulp and peel obtained with a hydroalcoholic solution at pH 2 were analysed by reversed-phase HPLC to identify six types of anthocyanins: cyanidin 3-glucoside, delphinidin 3-glucoside, pelargonidin 3-glucoside, cyanidin 3-o-glucoside, cyanidin 3,5-o-glucoside, and pelargonidin 3,5-o-glucoside [25]. Cyanidin 3-glucoside (12.67 mg/100 g), delphinidin 3-glucoside (0.82 mg/100 g) and pelargonidin 3-glucoside (1.76 mg/100 g) were the main anthocyanins in red pitaya.

Many researchers have also attempted to extract, purify, and detect polyphenols and beta-cyanins present in red pitaya using membrane filtration. Red pitaya fruits were passed through the ultrafiltration process using membranes with a 1 kDa molecular mass cut-off at 25 °C and pressure of 50 psi (nitrogen gas) to obtain clarified extracts, which were identified by UPLC-DAD-MS [31]. The ultrafiltration process recovered about 50% of the phenolic compounds and high levels of ferulic acid, caffeic acid, p-coumaric acid and quercetin were identified in the clarified extracts. In contrast, levels of gallic acid and resorcinol were lower in clarified extracts. This study indicated that membrane filtration coupled with chromatographic identification can be a potentially accurate option to identify and quantify the concentration leading to biological activity of the red pitaya fruit extracts.

### 3.2. Betalain

Betalains are a group of natural water-soluble nitrogen-containing pigments that originated from tyrosine in fruits and vegetables and are divided into two groups: beta-cyanins and betaxanthins [40]. Beta-cyanins in red pitaya contribute to the red-purple appearance and are involved in anti-inflammatory and antioxidant activities [22]. Song, et al. [41] reported that beta-cyanins in red pitaya had a significant effect on the alleviation of lipid accumulation as well as body weight gain induced by a high-fat diet. This was demonstrated by lowering the development of steatosis in the liver, total hepatic cholesterol, and triglyceride levels in mice. These observations were accompanied by increase of high-density lipoprotein (HDL) cholesterol in mice.

Generally, betalain or betacyanin were extracted and purified with different solvents under varied treatments prior to analysis, and the differences in the extraction conditions let to different yields, types, and concentrations. Chromatographic identification and quantification at different retention times and wavelengths were employed to analyze betalain extracts. The LC-MS data for peak identification of betalain in red pitaya fruits are shown in Table 3. García-Cruz, Dueñas, Santos-Buelgas, Valle-Guadarrama and Salinas-Moreno [16] extracted betalain using a solution of methanol: trifluoroacetic acid (1%) in water with the ratio of 80:20 (*v*/*v*) assisted with ultrasound for 30 min. The study identified 11 betalains of which nine were beta-cyanins, i.e., Gomphrenin I, Iso-gomphrenin I, 2-descarboxy-betanin, phyllocactin, 5-ο-(6′ο-3-hydroxybutyryl-*β*-glucoside, Isophyllocactin, 6′-ο-malonyl-e-descarboxy-betanin, a betanidin derivative, and 6′-ο-malonyl-e-descarboxy-isobetanin, the other two being betaxanthins (isoindicaxanthin and indicaxanthin). The betalain values ranged from 165.1 to 5423.4 mg/g dry weight. However, this study did not report on the purification of the extracted betalain, which might contain other polyphenols. In another study, red pitaya pulps were extracted by 60% methanol for 60 sec using a homogeniser [42]. The precipitation of pectic substances using ethanol was the first step in purifying beta-cyanins, followed by vacuum filtration with Whatman no. 4 paper. The extracts were then desalted with a C_18_-reversed phase cartridge followed by eluting with acidified methanol (95/5 methanol/pH 2 water, *v*/*v*). After rotary evaporation at 35 °C, the purified extract was dissolved in pH 5–6 water before analysis. There were seven peaks in that betacyanin chromatogram including betanin, iso-betanin, phyllocactin, isophyllocactin, betanidin 5-ο-(6′ο-3-hydroxybutyryl-*β*-glucoside and decarboxylated phyllocactin, with phyllocactin being the most abundant betacyanin. Notably, phyllocactin can be transformed into betanin via diacylation under high temperature and pH treatment [22]. Similarly, Fathordoobady, Mirhosseini, Selamat and Abd Manap [27] identified 12 types of betalain in both red pitaya peel and pulp, including betanidin 5-ο-*β*-sophoroside, betanin, iso-betanin, apiosyl-betanin, phyllocactin and others, listed in Table 3, by using supercritical fluid extraction.

Beta-cyanins have low stability in a food matrix, with water activity, light and temperature the crucial factors affecting their stability. A recent study analysed the stability of encapsulated beta-cyanins in alginate microbeads [43]. Beta-cyanins from oven-dried red pitaya peels were extracted using supercritical fluid extraction with CO_2_ and ethanol solution (10%) followed by micro-encapsulation. Similarly, betanin, phyllocactin and their isotypes were the major betalains in red pitaya peel, while it was revealed that the encapsulation approach improved the overall thermal, pH and storage stability of the betalain extract. Non-encapsulated beta-cyanins were lost when pH increased from 5 to 7 at temperatures higher than 60 °C.

### 3.3. Polysaccharides

#### 3.3.1. General Concepts

Polysaccharides are a group of long-chain polymeric carbohydrates composed of more than hundred monosaccharides bound with glycosidic linkages. Polysaccharides present in pitaya peel have attracted much attention in recent years for use in food products as thickeners, stabilisers and gelling agents [48,49]. Polysaccharides in red pitaya peel have been extracted and purified by various conventional (for example, heat reflux extraction) and novel extraction (e.g., ultra-high pressure enzyme) techniques in recent studies. The extraction methods are crucial in understanding the physicochemical characteristics and functional effects [50]. Additionally, structural mapping of monosaccharides is crucial to elucidate the structure and properties which are the building blocks of the polymer [51].

Heat reflux extraction was reported to decrease the bioactivity of purified polysaccharides due to the adverse effect of high temperatures, hence an ultra-high pressure enzymatic technique was used to extract polysaccharides from red pitaya peel [32]. Results showed that the main component of polysaccharides derived from red pitaya peel was a polysaccharide with 112.94 kDa average molecular mass, 17.19% degree of esterification, 97.22% water solubility, 5.51 g/g water holding capacity and 4.52 oil holding capacity. Galacturonic acid (42.56%), galactose (22.55%) and rhamnose (16.53%) were the main compositional monosaccharides. Furthermore, the study revealed that ultra-high pressure enzymatic extraction was more efficient than heat reflux extraction with higher extraction yield and better conservation of the polysaccharide bioactivity. Work by Montoya-Arroyo, et al. [52] on the characterisation of cell wall polysaccharides revealed the total pectic fraction constituted 50.23% of the cell wall of pitaya peel, with the major fraction being the water-soluble pectin fraction, followed by the oxalate-soluble pectin fraction. These could substitute for commercial gums in food and cosmetic products.

The polysaccharide structure obtained from white pitaya pulp was determined by Xu, et al. [53] using nuclear magnetic resonance spectroscopy. →4-*β*-D-GlcpA-1→, →6-*β*-D -Galp-1→ and →4-α-L-Rhap-1→ constituted the backbone of the polysaccharide with a molecular mass of 2.2 × 10^3^ kDa.

#### 3.3.2. Pectin

Red pitaya peel is one of the most important sources of pectin, a high-molecular-weight natural polysaccharide, accounting for up to 35% of cell walls [54]. Pectin is a group of galacturonic acid-rich polysaccharides and can be divided into two major groups, namely high methoxy and low methoxy pectin (higher and lower than 50% degree of esterification, respectively) [55]. Pectin is a natural gelling and thickening agent in the food industry used in fruit juice, fermented dairy products, jams and jellies [56]. Pectin utilisation begins with isolation from plant materials, and enzyme-assisted, subcritical water, microwave-assisted as well as ultrasound-assisted extractions have been reported to be the techniques of choice in recent years [55].

Thirugnanasambandham, et al. [57] extracted pectin from red pitaya peel using microwave-assisted methods (working frequency of 2450 MHz) at different solid-liquid ratios (95% ethanol), temperatures and extraction times. Maximum yield of pectin (7.5%) was detected at 45 °C, with an extraction time of 20 min and with a solid-liquid ratio of 24 g/mL. However, this study neither specified the temperature range, extraction times and solid-liquid ratios, nor analysed the physicochemical properties of the extracted pectin. Another study carried out by Muhammad, et al. [58] investigated the degree of esterification, molecular weight, and monosaccharide composition of extracted pectin from red pitaya peel. Pectin from the fresh inner layer of the red dragon peel appeared to be high methoxy, accounting for 63.74% of the degree of esterification, with 0.88 × 10^5^ Da molecular mass. Additionally, the study revealed that galacturonic acid was the predominant monosaccharide in pectin, constituting 39.11%, followed by mannose (17.78%) and rhamnose (14.47%). These results agreed with a recent study, in which different microwave intensities, ranging from 300 to 800 W, were employed for the extraction of pectin in red pitaya [59]. It was stated that galacturonic acid was the major monosaccharide, with the lowest degree of crystallinity being obtained at the lowest microwave intensity employed (300 W). The highest extraction yield of pectin was at 800 W microwave intensity owing to the damages in the middle lamella, leading to the disruption and collapse of cell walls.

### 3.4. Amino Acid Profiling

Amino acids are the basic units of protein, made up of amine (-NH_2_) and carboxyl (-COOH) functional groups with a side chain that determines the specific type of amino acid [60]. There are nine essential amino acids, namely histidine, isoleucine, leucine, lysine, methionine, phenylalanine, threonine, tryptophan and valine, that cannot be synthesized in the human body and must be obtained from foods [61]. Arivalagan, Karunakaran, Roy, Dinsha, Sindhu, Shilpashree, Satisha and Shivashankara [23] identified 18 amino acids in red pitaya (Table 4), eight of them being essential amino acids, using chromatography (UPLC-MS/MS). Results are in line with a study by Wu, Zhou, Zhang, Li, Jiang, Gao and Yun [49] reporting that phenylalanine (183 mg/g) is the most abundant amino acid in red pitaya. Both studies stated that red pitaya was a good source of essential amino acids, but the protein concentration in the fruit was much lower than the required nutrient reference values for daily intake.

### 3.5. Triterpenoids

Triterpenoids are another group of phytochemicals that are found in fruits and vegetables and exhibit antioxidant and anti-cancer effects [64]. Based on anticancer efficacy present in animal models, Bishayee, et al. [65] stated that triterpenoids derived from plants could be used as a cytotoxic agent against tumour cells to prevent breast cancer. The mechanism of cancer prohibition focuses on triterpenoids preventing cell proliferation and migration, along with the promotion of apoptosis (a process in human body that removes abnormal or unneeded cells), leading to the cleavage of cancer cells [66].

Red pitaya extracts were prepared using supercritical carbon dioxide, and identified by gas chromatography-mass spectroscopy [67]. Results showed that red pitaya, predominantly composed of *β*-amyrin (15.87%), α-amyrin (13.90%), octa-cosane (12.2%) and γ-sitosterol (9.35%), contained higher concentrations (29.77% of the total composition) of triterpenoids than that of white pitaya (23.39% of the total composition). Further studies on triterpenoids in pitaya are needed to determine optimum extraction conditions, compositions, and bioactive effects.

## 4. Antioxidant Activity

### 4.1. Glycaemic Control

Blood glucose levels reflect the amount of glucose released into the blood stream following food consumption, which is an important parameter to measure, since high blood glucose levels can cause diabetes, high blood pressure and other chronic diseases [68]. Red pitaya is a potential medicinal plant for attenuation of blood glucose due to the regeneration of pancreatic *β*-cells that synthesise and secrete insulin and amylin (two hormones that regulate blood glucose levels) [4]. There is growing evidence to suggest that beta-cyanins derived from red pitaya have a significant effect on attenuating blood glucose and preventing fatty liver, as well as cardiovascular disease.

A study investigated the effect of red pitaya on lipid metabolism and glycaemia, in which mice were treated with different diets, namely standard diet, hyper-cholesteraemic diet, lipid-lowering diet and red pitaya pulp diet (100, 200 and 400 mg/kg) [47]. Results showed that the total cholesterol and blood glucose level in red pitaya treated animals significantly decreased compared to their counterparts due to the presence of betalains, oligosaccharides, quercetin, and other bioactive compounds. However, the mechanism contributing to hypo-cholesterolaemia and blood glucose modulation was not discussed in detail.

Song, Chu, Yan, Yang, Han and Zheng [41] isolated beta-cyanins from red pitaya, which were orally administrated to high-fat diet mice (200 mg/kg/day) for 14 weeks. The betacyanin incorporating diet significantly reduced mice body weight gain, although no difference was observed in the daily calorie intake between the betacyanin treated and high-fat treated diet mice. Lower blood glucose and insulin levels were detected in the pitaya betacyanin group, in addition to the modulation of gut microbiota in mice. They showed a decrease in the content of *Firmicutes* and *Bacteroidetes* and an increase in the proportion of *Akkermansia*, which safeguards the healthy state of the human gastrointestinal tract by regulating host energy balance. It was indicated that understanding how betacyanin from red pitaya fruit targets the gut microbiota is an area of research that needs expanding to understand the mechanism involved in these health benefits. This was supported by the observation that pitaya betacyanin lowered serum triglyceride, total cholesterol and LDL cholesterol but increased HDL cholesterol levels couple, with an overall improvement of the lipid profile. This study is in line with the findings by Lugo-Radillo, et al. [69], arguing that betanidin reduces the glycaemic response in mice compared to the mice fed with an atherogenic diet, accounting for 50.94%, and limits the expression of quinone reductases and activity of DNA methyltransferase. It appears that betacyanin present in red pitaya fruit is an important component for the regulation of blood glucose levels, enhancement of lipid profile, and prevention of lipid accumulation. The antidiabetic effect of beta-cyanins derived from red pitaya is strongly correlated with betacyanin-starch and betacyanin-amylase interactions, which require further investigation.

### 4.2. Anti-Inflammatory Activity

Inflammation is a defensive response of the immune system to harmful bacteria and viruses in order to combat infections and repair tissues and wounds. It is the main cause to different human diseases, including cancer, type II diabetes, and cardiovascular diseases [70]. Scientific evidence has reported that polyphenols and flavonoids derived from fruits and vegetables exhibit strong anti-inflammatory activity and decrease the formation of reactive oxygen species (ROS) [71,72].

A study demonstrated that betalains from red pitaya fruit peel showed up to 92.06% of irritation inhibition on the sodium dodecyl sulphate-induced vascular irritation of duck embryo chorioallantoic membrane due to the strong scavenging ability of free radicals [73]. In another study, Saenjum, Pattananandecha and Nakagawa [25] determined the inhibitory effects on both reactive oxygen and nitrogen species to analyse the anti-inflammatory activities of red pitaya. The cell-based study reported that the fresh red pulp extract (containing anthocyanin and catechin) had the highest inhibition of both reactive oxygen and nitrogen species, and higher numbers of free hydroxyl groups around the pyrone ring of anthocyanin positively affected the antioxidant capacity. The concentration of 25–100 mg/L showed active inhibitory effect on both reactive species. It was also reported that the cyanidin 3-glucoside suppressed the generation of tumour necrosis factor and modulated gene expression. Montiel-Sánchez, et al. [74] observed a similar result and stated that the extracts from red pitaya pulp showed higher in vitro anti-inflammatory activity, representing 82.81%, and betalains were the main effective phytochemicals.

### 4.3. Other Bioactivities

The polyphenol extracts of red pitaya with high anti-microbial activity were reported by some researchers. Zambrano, et al. [75] studied the anti-microbial activity of pitaya extracts (phenolic compounds) against foodborne pathogens and spoilage bacteria. They found that the growth of *Bacillus subtilis*, *Staphylococcus aureus* and *Salmonella enterica* were significantly inhibited compared with *Listeria monocytogenes* and *Pseudomonas* strains. Except for phenolics, beta-cyanins were reported to control the reproduction of the *S. aureus*, *Enterococcus* spp. and *Bacillus* spp. with an antimicrobial activity (MIC) range from 12.5–25 mg/mL [76].

Anti-mutagenicity of betacyanin extracts was studied by Thaiudom, Oonsivilai and Thaiwong [26] and *Salmonella typhimurium* TA98 was used as the test strain. The results revealed that the colony amounts of *S. typhimurium* TA98 decreased with the addition of betacyanin extracts and inhibited the mutation. IC_50_ of the antimutagenicity activity was observed at 0.522 mg GAE/mL of the total phenolic content. However, this study did not explain the inhibitory mechanism and did not deal with safe use in human diet.

Collectively, many researchers have confirmed the bioactivities, including anti-hyper-glycaemia, anti-inflammatory, anti-microbial and anti-mutagenicity, of the polyphenol extracts from red pitaya, but the mechanism has yet to be elucidated.

## 5. Red Pitaya Utilisation in Food Products

### 5.1. Wheat Products

**Figure 2:**
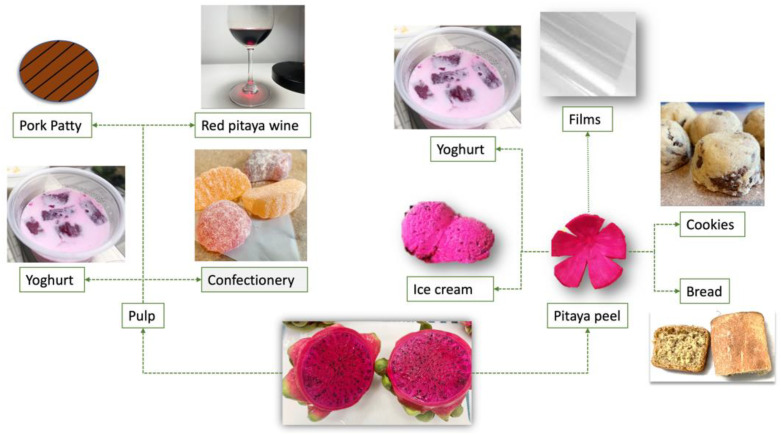
Utilisation of red pitaya in food products.

Wheat is a basic ingredient in many food products, including bread, cookies, noodles and pasta. Wheat flour can form an elastic dough when it is mixed with water due to the presence of two protein fractions of gluten, namely glutenin and gliadin [77]. This, and the incorporation of fruit dietary fibre from red pitaya fruit in traditional wheat based products, has been studied in recent years due to increasing health conscious consumers [78]; the utilisation of red pitaya in various food product is summarised in Figure 2.

Hsu, et al. [79] assessed the changes in colour, texture, betacyanin content, free phenolic content and antioxidant capacity of Chinese steamed bread and dough containing red pitaya peel. The dough was prepared by adding 0%, 3%, 6% and 9% of the air-dried red pitaya peel powder to the formulation. Addition of red pitaya peel to bread dough resulted in higher values of redness and colour acceptability compared to plain wheat dough, whereas the extensibility and moisture content decreased due to the dilution of gluten proteins and fibre-water interactions. The enrichment also improved the overall antioxidant capacity and free phenolic acid content in both bread and dough. Sensory evaluation showed that steam bread enriched with red pitaya peel powder was not as desirable as plain steam bread, achieving lower organoleptic scores on odour and texture.

Another study evaluated the nutritional composition and physical properties of cookies made with different proportions of white pitaya peel powder (5%, 10% and 15%) and concluded that its introduction reduced moisture content, water activity and thickness, as compared to the original formulation. Pitaya powder did not have a significant impact on protein or fat content, hardness, texture or aroma in the cookies [80]. An increase in redness and a decrease in yellowness were closely associated with the presence of pitaya peel powder in cookies. Results were supported by Shiau, et al. [81], who reported that noodles fortified with 3% to 9% pitaya peel powder exhibited a markedly higher betalain and flavonoid content as well as radical scavenging ability compared to plain wheat noodles, but the cooking process affected the betalain content and the redness of materials.

### 5.2. Dairy Products

Numerous studies focused on the utilisation of prebiotic oligosaccharides and potential applications of the colourants from red pitaya fruit in dairy products, such as yoghurt and milk. Zainoldin and Baba [82] studied the effects of red and white pitaya on the nutritional properties and antioxidant capacity of yoghurt. Results demonstrated that the fermentation rate of milk was improved by the addition of flesh from red and white pitaya by decreasing the pH value. White pitaya enriched yoghurt had a higher lactic acid content and fermentation rate compared to red pitaya yoghurt. Yoghurt enriched by both pitaya fruits had better syneresis than plain yoghurt since the water-holding capability of the former enhanced the content of whey into yoghurt. Furthermore, the phenolic content of yoghurt with white pitaya was higher than for red pitaya yoghurt, whereas the latter showed a higher radical scavenging ability. The peptide concentration present in pitaya incorporated yoghurt remained constant in comparison to plain yoghurt due to the low protein content in pitaya fruit. Results were in accordance with Tze, et al. [83], who reported that red pitaya fruit powder only had 0.182% protein. A biochemical test carried out by Yien Ong, et al. [84], confirmed that a high level of lactic acid bacteria was present in fermented red pitaya juice, indicating that the incorporation of red pitaya in yoghurts can increase the acidity and number of probiotic microorganisms.

Lima, et al. [85] studied the metabolic profile and potential applications of microfiltered red-purple pitaya colourant in yoghurt using UPLC-ESI-QTOF-MS. Yoghurt was prepared with 0.5%, 1.0%, 1.5% and 2% of the microfiltered red pitaya peel, and the nutritional profile, sensory analysis, microbiological analysis, and storage stability for twelve weeks were assessed. During storage, the pH and soluble solids content remained constant at about 3.90 and 63 °Brix, respectively, while the degradation of phenolic compounds (52%) occurred after four weeks. Yoghurts with red pitaya peel exhibited high yeast and mould counts in the twelfth week. The sensory analysis results showed that the incorporation of red pitaya peel into the yoghurts did not alter the aroma. However, it did enhance the colour and visual appearance, showing that red pitaya peel can be used as a natural ingredient for the enhancement of colour quality in food products.

Red pitaya peel fibre was utilised as a fat replacer in ice cream made from whole milk or skim milk powder, assessing physiochemical properties that included melting rate, texture, colour, betacyanin content, antioxidant activity and sensory profile [12]. Results showed that addition of red pitaya peel powder yielded high total dietary fibre, protein content, minerals and antioxidant capacity in ice cream with whole milk powder, contributing to an increase in water retention capacity and resistance to melting. A 73.5% decrease in lipid content was observed in ice cream enriched with red pitaya peel powder, together with an improvement in overrun and texture.

### 5.3. Confectionery

Confectionery is a group of food products rich in sugar and carbohydrates, including gummies, marshmallows and hard candies [86]. Nutritional enrichment with natural compounds from fruit and vegetable has been a recent consumer trend. Betalains from red pitaya fruit are widely used as natural colour and antioxidant in food products due to their red-violet colour as well as high radical scavenging ability [87]. Hani, et al. [88] found that red pitaya puree provided good texture and antioxidant capacity in gummy confections that supports it use as a vibrant red colourant. Gummy confections were prepared using fish gelatine and high-methoxy pectin as gelling agents in the presence of red pitaya puree (20–25%). The hardness of gummies with 20% puree increased from 13.49 to 17.93 kg with a higher content of fish gelatine and high-methoxy pectin, while hardness decreased in the 25% gummies, an outcome which merits further investigation. In single biopolymer systems, the hardness and gumminess in samples made of gelatine decreased with the incorporation of red pitaya puree due to a certain heterogeneity in the polymeric network, which was also less ordered. The hardness in gummies with high-methoxy pectin did not show a significant variation upon addition of red pitaya puree due to microphase separation between fruit puree-rich and polymer-rich phases. Red pitaya puree enriched gummies appeared to have high bioactivity, water content and redness. It appears that gelatine preserves betalain pigment well in gummies, with no adverse effect on texture and other sensory properties.

### 5.4. Wine

Grape wine has been consumed worldwide for centuries, and wine fermented from fruits, i.e., apples, pears and red pitaya, and vegetables, has emerged as a promising approach to enhance sensory and nutritional properties [89]. Pitaya fruit contains high levels of pectin in both peel and pulp, and pectolytic enzymes could be used to increase juice yield before wine fermentation. Such pectinase effect prior to the inoculation with *T. delbrueckii* yeast has been demonstrated [90]. Pectinase treatments showed no significant difference in alcoholic fermentation, total soluble solid concentration, composition of organic acids, except for acetic acid (higher production), or other basic oenological properties. However, the treatment increased the yield of juice and viscosity and red dragon fruit wines had high levels of total volatile compounds, including alcohols, esters and terpenes. Although the application of pectinase increases the total phenolic content, betacyanin concentration decreases with pectinase treatment. This is due to *β*-glucosidase in the yeast hydrolysing glycosidic bonds in glycosylated beta-cyanins to release unstable betanidin. The lightness and redness of red pitaya wines decreased with the application of pectinase.

Another study analysed the effect of different yeast strains, namely *Saccharomyces cerevisiae* EC-118, *Torulaspora delbrueckii Biodiva* and *Lachancea thermotolerans Concerto*, on the physicochemical properties of red pitaya wines [91]. Changes in total soluble solids content showed the same patterns in all three yeast strains, i.e., decreasing in the first two days and then remaining constant until day 8–10, with final °Brix value, ethanol level and total sugar residues in all treatments being between 8.3–8.6, 8.3–8.9% and lower than 2.0 g/L, respectively. Red pitaya wine with *S. cerevisiae* contained the highest glycerol level (9.60 g/L) while *L. thermotolerans* significantly increased the lactic acid (1.8 g/L) and succinic acid (1.67 g/L) content. *T. delbrueckii* fermented red pitaya wine retained the highest number of amino containing compounds, including alanine (6.55 mg/L), ammonium (7.50 mg/L) and arginine (4.95 mg/L), over wine fermented with the other two strains. Regarding volatiles, *T. delbrueckii* fermented wines exhibited the highest level of terpenes, isoamyl acetate (esters), isoamyl alcohol and 2-phenylethyl alcohol, while *S. cerevisiae* produced the highest level of total ethyl octanoate and ethyl decanoate. Overall, the study concluded that *T. delbrueckii* and *L. thermotolerans* were the best yeasts for optimal red pitaya wine production, but it did not compare the content and variety of major volatiles with grape wine fermented with the same yeast strains.

### 5.5. Meat Products

The composition of meat varies among different types of animals and mainly comprises water, proteins, polyunsaturated fatty acids, essential amino acids and lipids. Oxidation of lipids is one of the major causes of deterioration of meat products, leading to changes in colour, flavour, texture and nutritional properties [92]. Utilisation of natural antioxidants derived from fruits and vegetables has been studied for potential applications in meat products to extend shelf-life and prevent lipid oxidation and spoilage. Freeze-dried red pitaya pulp powder was added to pork patties at three concentrations (250, 500 and 1000 mg/kg) according to the method reported by Bellucci, et al. [93]. Cooking loss, texture and pH of pork patties did not show significant differences with or without red pitaya powder in the formulation. In contrast, antioxidant capacity increased in the pitaya fruit enriched pork patty and a lower rate of lipid oxidation was obtained (from 2.44 to 1.13 mg/kg), owing to the presence of beta-cyanins. Oleic acid (53.02–55.48%), palmitic acid (16.21–18.36%), stearic acid (8.79–9.41%) and linoleic acid (10.43–12.71%) were the predominant monounsaturated, saturated and polyunsaturated fatty acids in pork patties with red pitaya powder. This type of incorporation lowered the content of saturated fatty acids but increased the concentration of unsaturated fatty acids in the blood. It was suggested that the red pitaya extract could be used in meat products to improve sensory properties and prolong shelf-life.

### 5.6. Packaging Films

Bio-degradable packaging films derived from plants have received increasing attention for the development of environmentally friendly materials [94]. Polysaccharides, proteins and starch show promise as film-forming materials, exhibiting efficient preservation of food nutrients and an extended shelf life [95].

Qin, et al. [96] compared the effects of different contents (0.25, 0.50 and 1.00%) derived from red pitaya peel on starch and polyvinyl alcohol films. They reported that the total betacyanin content was 56 mg/100 g in fresh peel, with natural pH 3.85 in water facilitating the formation of compact film cross-sections, as the extracts were incorporated well in the film matrix. Beta-cyanins interacted with starch, polyvinyl alcohol and glycerol through hydrogen bonds based on the intensity and position of oxygen and hydrogen stretching bands in FTIR (Fourier Transform Infrared) spectra. Betacyanin-rich samples also showed high antioxidant and antimicrobial potential against foodborne pathogens, including *Staphylococcus aureus*, *Listeria monocytogenes*, *Escherichia coli* and *Salmonella*.

## 6. Conclusions

This review summarises the polyphenol and polysaccharide profiles in red pitaya, extraction techniques, pitaya fruit maturation, glycaemic modulation, and utilisation in food products. During the growth and maturation period of red pitaya, the betalain content increased dramatically during later development, along with the reduction of betaxanthin in the peel. Red pitaya seed was reported to have higher amounts of total phenolic acids and flavonoids over peel and pulp, while pulp contained higher levels of cyanidin 3-glucoside, delphinidin 3-glucoside and pelargonidin 3-glucoside. Caffeic acid, ferulic acid protocatechuic acid and p-coumaric acid were quantified as the dominant polyphenols. Betanin, iso-betanin, phyllo-cactin and isophyllocactin were the major beta-cyanins identified in red pitaya fruit. There are eight essential amino acids, predominantly composed of phenylalanine and arginine, and four triterpenoids (*β*-amyrin, α-amyrin, octacosane and γ-sitosterol) identified in red pitaya. The bioactive compounds, especially betacyanin, in red pitaya fruits have been found to improve lipid profile, regulate glycaemic response, mitigate diabetes, and modulate the immune system, but the studies are limited, and mechanisms are yet to be understood. It has been found that polysaccharide purified from red pitaya can be used as a natural gum and stabiliser in dairy products and confectionary to increase water-holding capability and decrease hardness. The enrichment of red pitaya in wheat products was reported to alter physical parameters (i.e., redness and hardness) with the increase of bioactive compounds. Further work is required to discover the optimal purification methods for individual polyphenols or polysaccharides, and their effects on human health and utilisation in food products, besides using whole red pitaya fruits.

## Figures and Tables

**Table 1:** Extraction of polyphenols from red pitaya.

Extraction Methods	Compounds	Quantification Methods	Concentrations (Dry Weight)	Reference
Freeze-dried fruit pulps were mixed with 80% ethanol (1:2) in an ultrasonic bath in the dark for 30 min at 60 °C followed by centrifugation at 5000× *g* for 15 min. The supernatant was collected, and ethanol was evaporated by a rotary evaporator.	Phenolics	Folin-Ciocalteu reagent	48.30 ± 0.10 mg GAE/100 g	[23]
	Flavonoids	Aluminium chloride method (Catechin was used as a standard)	31.2 ± 1.30 mg CE/100 g	
	Beta-cyanins	McIlvaine buffer method (at 538 nm)	20.4 ± 1.90 mg BCE/100 g	
A microwave-assisted extraction with different ratios of solid/water, from 1/50–1/150 g/mL, at 600 W for 5–65 min) was used to extract phenolic compounds and beta-cyanins in freeze-dried pulps followed by centrifuged twice at 4000× *g* for 10 min each time.	Phenolics	HPLC-DAD-ESI/MS	2.39–10.14 mg/g	[24]
	Flavonoids	HPLC-DAD	3.22–5.04 mg/g	
	Beta-cyanins	HPLC-DAD	0.43–1.66 mg/g	
Freeze-dried peels were mixed with different proportions of ethanol (from 30% to 60%) at varied solvent/solid ratios (10:1–30:1) in an ultrasonic bath in the dark for 5–25 min at different temperatures (30–70 °C).	Phenolics	Folin-Ciocalteu reagent	4.51–8.54 mg GAE/g	[2]
	Beta-cyanins	McIlvaine buffer method (at 537 nm absorption)	1.02–1.60 mg BCE/g	
Freeze-dried peels were homogenized with deionized water at pH 5.5, 40 °C for 20 min. The extract was kept in the dark in a refrigerator until analysis.	Phenolics	Folin-Ciocalteu reagent	35.81 ± 3.11 mg GAE/g	[26]
	Beta-cyanins	Liquid Chromatography Mass Selective Detector (LCMS)	15.21 ± 0.04 mg/g	
A comparison of solvent extraction and supercritical fluid extraction was conducted. Oven-dried pitaya peel or flesh (5 g) were extracted with 50 mL ethanol solvent (100%, 90%, 70%, 50%, 30%, 10% and 0%) for 20 min at 25 °C with a magnetic stirrer at 300 RPM followed by filtrated through a Whatman No. 4 paper. Samples (5 g) were treated with a supercritical fluid extraction system with 99.9% purity of CO_2_ at 2 mL/min flow rate and 10% ethanol solvent (50 mL). The extraction was conducted under 25 MPa pressure and 50 °C.	Phenolics	Folin-Ciocalteu agent	139 ± 0.41 (peel, 50% ethanol), 105 ± 0.53 (flesh, 50% ethanol), 135.75 (peel, supercritical fluid) and 99.42 (flesh, supercritical fluid) GAE/100 g	[27]
	Beta-cyanins	McIlvaine buffer method (at 538 nm)	28.44 ± 1.98 (peel, 50% ethanol), 84.25 ± 0.89 (flesh, 50% ethanol), 24.58 ± 2.16 (peel, supercritical fluid), and 91.20 ± 2.23 (flesh, supercritical fluid) mg/100 mL	
The juice was extracted by a juice extractor and filtered through a hand-held kitchen sieve followed by centrifugation at 3000× *g* for 10 min at 4 °C. The supernatant was vacuum filtered through a filter paper (F1001, CHMLAB Group) and stored at −20 °C until analysis.	Phenolics	Folin-Ciocalteu agent	17.80 ± 0.70 mg GAE/100 g	[28]
	Beta-cyanins	McIlvaine buffer method (at 537 nm)	0.20 ± 0.01 mg BCE/L	
Freeze-dried red pitaya peel, pulp and seed were mixed in a hydroalcoholic solution at pH 2 to obtain the extracts. The extracts were filtrated, and the solvent was evaporated by vacuum drying under reduced pressure.	Phenolics	Folin-Ciocalteu agent	294.8 ± 12.9 (peel), 277.6 ± 14.2 (pulp) and 375.1 ± 12.6 (seed) mg GAE/g	[25]
	Flavonoids	Aluminium chloride method (quercetin was used as a standard)	193.8 ± 11.7 (peel), 177.4 ± 12.5 (pulp), and 264.4 ± 10.8 (seed) mg QE/g	
	Anthocyanin	Modified pH differential method (cyanidin chloride as positive control)	135.4 ± 9.3 (peel) and 159.7 ± 8.9 (pulp) mg CCE/g	
Oven-dried peels or seeds (0.5 g) were homogenized with 80% methanol (15 mL) on ice for 15 min using Ultra-Turrax homogeniser followed by centrifugation at 4000× *g* for 15 min at 4 °C. The supernatant was re-extracted twice before store in a freezer at −20 °C and covered with aluminium foil.	Phenolics	Folin-Ciocalteu reagent	0.18 ± 0.01 and 0.31 ± 0.02 g/100 g	[29]
Air-dried seed powder ( <0.85 mm particle-sized) were extracted by using 90% ethanol (1:10) in an Innova 4000 incubator shaker for 24 h at 30 °C and 150 rpm. The extracts were filtrated through Whatman No. 4 filter paper followed by evaporation at 40 °C to remove solvents.	Phenolics	Folin-Ciocalteu reagent	13.56 ± 2.04 mg/g	[30]
	Catechin	HPLC	3.60 ± 2.33 mg/g	

**Table 2:** Identification of phenolics and flavonoids present in red pitaya.

Polyphenols	Chemical Structure	Molecular Formula	Concentrations (Dry Weight)	References
Phenolic compounds				
Caffeic acid	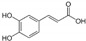	C_9_H_8_O_4_	20.1 ± 19.6 mg/100 g	[23,32]
Ferulic acid	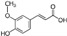	C_10_H_10_O_4_	14.37 ± 9.3 mg/100 g	[33]
Protocatechuic acid	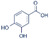	C_7_H_6_O_4_	10.32 ± 4.8 mg/100 g	[34]
p-Coumaric acid	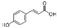	C_9_H_8_O_3_	1.52 ± 1.4 mg/100 g	[35]
Sinapic acid	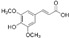	C_11_H_12_O_5_	1.12 ± 0.8 mg/100 g	[23]
Vanillic acid	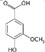	C_8_H_8_O_4_	0.918 ± 0.4 mg/100 g	[23]
Gallic acid	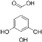	C_7_H_6_O_5_	159.7 ± 19.7 µg/100 g	[36]
o-Coumaric acid	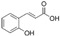	C_9_H_8_O_3_	199.4 ± 49.5 µg/100 g	[23]
t-Cinnamic acid	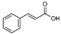	C_9_H_8_O_2_	51.7 ± 44.1 µg/100 g	[23]
Salicylic acid	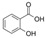	C_7_H_6_O_3_	50.2 ± 20.3 µg/100 g	[23]
Syringic acid	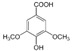	C_9_H_10_O_5_	65.10 ± 0.04 µg/100 g	[37]
Flavonoids				
Catechin	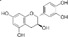	C_15_H_14_O_6_	3.60 ± 2.33 mg/g	[25]
Quercetin	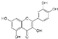	C_15_H_10_O_7_	1.31 ± 0.45 mg/g	[25]
Epicatechin	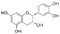	C_15_H_14_O_6_	30.77 ± 0.04 µg/100 g	[37]
Epigallocatechin gallate	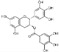	C_22_H_18_O_11_	58.99 ± 0.01 µg/100 g	[38]
Procyanidin B1	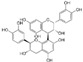	C_30_H_26_O_12_	1325.61 ± 0.07 µg/100 g	[39]
Procyanidin B2	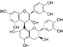	C_30_H_26_O_12_	589.26 ± 0.03 µg/100 g	[39]
Kaempferol 3-glucoside	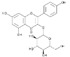	C_21_H_20_O_11_	39.69 ± 0.02 µg/100 g	[37]
Quercetin 3-glucoside	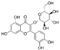	C_21_H_20_O_12_	31.93 ± 0.04 µg/100 g	[37]
Rutin	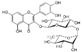	C_27_H_30_O_16_	26.57 ± 0.04 µg/100 g	[37]
Anthocyanins				
Cyanidin 3-glucoside	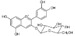	C_21_H_21_O_11_	12.67 ± 0.63 mg/100 g	[25]
Delphinidin 3-glucoside	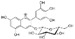	C_21_H_21_ClO_12_	0.82 ± 0.17 mg/100 g	[25]
Pelargonidin 3-glucoside	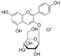	C_21_H_21_O_10_	1.76 ± 0.23 mg/100 g	[25]

**Table 3:** LC-MS data for peak identification of betalains in red pitaya fruit.

Beta-cyanins	Retention Time (min)	Molecular Formula	λ_max_ (nm)	[M + H]^+^ (*m*/*z*)	MS^2^ (*m*/*z*)	Reference
Betanidin 5-ο-*β*-sophoroside	3.9	C_30_H_36_N_2_O_18_	NR	713	551, 389	[27]
Apiosyl-betanin	10.1	C_29_H_36_N_2_O_18_	NR	683	551.389	[27]
Butyryl-betanin	11.0	C_27_H_28_N_2_O_16_	NR	637	551, 389	[42]
Betanidin-5-ο-*β*-glucoside (Betanin)	11.5	C_24_H_26_N_2_O_13_	535	551.1	389,343	[44]
Hylocerenin (3-hydroxy-3-methyl-glutaryl-betanin)	11.9	C_30_H_27_N_2_O_17_	NR	695	551, 389	[27]
Iso-Butyryl-betanin	12.5	C_27_H_28_N_2_O_16_	NR	637	551, 389	[27]
2′-Apiosyl-phyllocactin	12.9	C_32_H_40_N_2_O_20_	NR	769	683, 551, 389	[27]
Isohylocerenin	13.7	C_30_H_27_N_2_O_17_	NR	695	551, 389	[27]
2′-Apiosyl-isophyllocactin	13.8	C_32_H_40_N_2_O_20_	NR	769	683, 551, 389	[27]
Iso-betanidin 5-ο-*β*-glucoside (Iso-betanin)	16.9	C_24_H_26_N_2_O_13_	535	551.1	Not reported	[42]
17-decarboxy-betanin	18.28	C_23_H_27_N_2_O_11_	505	507.16	389.10, 343.09	[45]
Gomphrenin I	19.6	C_24_H_26_N_2_O_13_	536	551	389	[16]
Isogomphrenin I	21.1	C_24_H_26_N_2_O_13_	538	551	389	[16]
2-descarboxy-betanin	22.7	C_17_H_17_N_2_O_6_	498	507	345	[16]
Phyllocactin	23.1	C_27_H_29_N_2_O_16_	536	637	593, 551, 389	[46]
Isophyllocactin	24.2	C_27_H_28_N_2_O_16_	538	637	593, 551, 389	[47]

**Table 4:** Monosaccharide and amino acid profiles of red pitaya.

Name	Chemical Structure	Molecular Formula	Molecular Mass (g/mol)	Composition	References
Monosaccharide					
Mannose	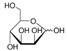	C_6_H_12_O_6_	180.16	17.78 ± 1.07%	[58]
Rhamnose	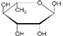	C_6_H_12_O_5_	164.16	14.47 ± 0.39%	[62]
Galacturonic acid	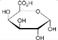	C_6_H_10_O_7_	194.14	39.11 ± 1.87%	[58]
Glucose	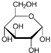	C_6_H_18_O_6_	180.16	10.82 ± 0.44%	[58]
Galactose	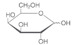	C_6_H_12_O_6_	180.16	11.91 ± 0.48%	[58]
Xylose	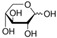	C_5_H_10_O_5_	150.13	2.41 ± 0.02%	[58]
Arabinose	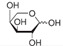	C_5_H_10_O_5_	150.13	3.49 ± 0.07%	[62]
Fructose	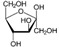	C_6_H2_0_O_6_	180.16	1.10–4.30 g/100 g	[52]
Sorbose	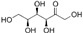	C_6_H_12_O_6_	180.16	1.87 mg/100 g	[49]
Arabino-furanose	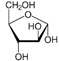	C_5_H_10_O_5_	150.13	0.75 mg/100 g	[49]
Maltose	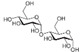	C_12_H_22_O_11_	342.30	1.02 mg/100 g	[49]
Amino Acids					
Histidine	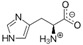	C_6_H_9_N_3_O_2_	155.15	36.3 ± 3.0 mg/g	[23]
Leucine	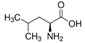	C_6_H_13_NO_2_	131.17	38.1 ± 1.4 mg/g	[23]
Lysine	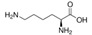	C_6_H_14_N_2_O_2_	146.19	72.8 ± 2.4 mg/g	[23]
Methionine	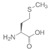	C_5_H_11_NO_2_S	149.21	30.6 ± 3.9 mg/g	[23]
Cystine	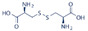	C_6_H_12_N_2_O_4_S_2_	240.30	1.66 ± 0.2 mg/g	[23]
phenylalanine	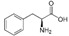	C_9_H_11_NO_2_	165.19	183 ± 4.1 mg/g	[23]
Tyrosine	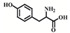	C_9_H_11_NO_3_	181.19	65.5 ± 2.2 mg/g	[63]
Tryptophan	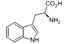	C_11_H_12_N_2_O_2_	204.23	0.055 ± 0.01 mg/g	[23]
Valine	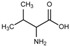	C_5_H_11_NO_2_	117.15	32.5 ± 0.6 mg/g	[23]
Glycine	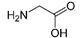	C_2_H_6_NO_2_	75.07	0.259 ± 0.03 mg/g	[23]
Alanine	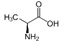	C_3_H_7_NO_2_	89.09	35.4 ± 1.3 mg/g	[23]
Serine	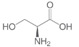	C_3_H_7_NO_3_	105.09	42.0 ± 8.5 mg/g	[49]
Proline	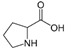	C_5_H_9_NO_2_	115.13	83.1 ± 4.8 mg/g	[49]
Threonine	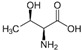	C_4_H_9_NO_3_	119.12	28.2 ± 2.0 mg/g	[23]
Asparagine	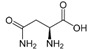	C_4_H_8_N_2_O_3_	132.12	6.60 ± 1.3 mg/g	[23]
Aspartic acid	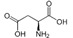	C_4_H_7_NO_4_	133.11	47.8 ± 6.4 mg/g	[23,49]
Glutamic acid	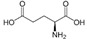	C_5_H_9_NO_4_	147.13	127 ± 4.6 mg/g	[23,49]
Arginine	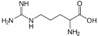	C_6_H_14_N_4_O_2_	174.2	149 ± 3.8 mg/g	[23,49]

## Data Availability

The original contributions generated for this study are included in the article; the data presented in this study are available on request from the authors.
